# The Prognostic Impact of Lymph Node Involvement in Large Scale Operable Node-Positive Esophageal Squamous Cell Carcinoma Patients: A 10-Year Experience

**DOI:** 10.1371/journal.pone.0133076

**Published:** 2015-07-15

**Authors:** Xiao-Ling Xu, Wei-Hui Zheng, Shuang-Mei Zhu, An Zhao, Wei-Min Mao

**Affiliations:** 1 Department of Medical Oncology, Zhejiang Cancer Hospital, 38 Guangji Road, Hangzhou City, China; 2 Key Laboratory on Diagnosis and Treatment Technology on Thoracic Cancer, Zhejiang Cancer Hospital (Zhejiang Cancer Research Institute), Hangzhou, Zhejiang Province, China; 3 Department of Radio-Chemotherapy Oncology, Lishui People’s Hospital, The Sixth Affiliated Hospital of Wenzhou Medical University, Wenzhou, Zhejiang, China; 4 Department of Thoracic Surgery, Zhejiang Cancer Hospital, Hangzhou, Zhejiang Province, China; Chinese Academy of Medical Sciences, CHINA

## Abstract

**Background:**

Lymph node (LN)-related factors including the number of LN regions involved, the LN ratio (LNR), and the number of metastatic LNs are strong prognostic indicators for esophageal squamous cell carcinoma (ESCC) patients. Accurately staging LN involvement may improve the stratification of patients and guide the management of patients.

**Methods:**

A total of 688 potentially resectable patients who had regional LN metastases were enrolled in this retrospective study.

**Results:**

ESCC involving a single region was associated with better outcomes than that involving multiple regions (*P* < 0.001 for both PFS and OS). An increased number of metastatic LNs was significantly associated with reduced PFS and OS based on univariate analysis (*P* < 0.001). PFS and OS were significantly higher in patients with a lower cancer-involved LNR, with 5-year OS rates of 9.7% and 31.4% for patients with a lower and higher cancer-involved LNR, respectively. Based on multivariate analysis, patients with N1 LN involvement experienced longer survival than patients with N2 LN involvement (HR: 1.37; 95% CI: 1.12-1.68) or N3 LN involvement (HR: 1.96; 95% CI: 1.52-2.53). Higher LNR resulted in longer OS than lower LNR based on multivariate analysis (HR: 1.45; 95% CI: 1.15-1.84; *P* = 0.002).

**Conclusions:**

Our study has shown that not only the number of metastatic LNs but also the number of involved LN regions predicts outcomes after definitive surgery among Chinese patients with N-positive ESCC. LNR might serve as a powerful indicator that should be included in TNM staging for EC patients.

## Introduction

Although there have been some improvements in the diagnosis and therapy of esophageal cancer (EC), it remains one of the leading causes of cancer-related mortality worldwide, resulting in 406,800 deaths annually [1]. Esophageal squamous cell carcinoma (ESCC) is the most common type of EC in Asia, especially in China [1]. The prognosis of EC patients remains poor, with a 5-year overall survival (OS) rate of less than 37% [2, 3]. Lymph node (LN) metastasis is one of most important prognostic factors of EC. The 5-year OS rate after surgical resection is 18–47% for patients with LN metastasis, which is significantly less than that for patients without nodal involvement [4, 5].

According to previous studies, node-positive status is found in 47.3–61.8% of patients with resected ESCC [3, 6]. Accurately staging LN involvement plays a critical role in the management of patients with EC. The American Joint Committee on Cancer (AJCC)/Union International Against Cancer (UICC) tumor node metastasis (TNM) cancer staging system has widely been used to stratify EC patients and to choose optimal treatment strategies. The 7th and most recent edition of the AJCC/UICC TNM classification was released in late 2009 [7]. In this edition, N is defined as the number of regional LNs involved (N0, 0 nodes; N1, 1 to 2 nodes; N2, 3 to 6 nodes; and N3, more than 7 nodes). However, according to the 6th edition of the AJCC/UICC TNM classification, N staging can be subclassified as the absence (N0) or presence (N1) of paraesophageal LN involvement in EC patients. The treatment practice for those patients with locally advanced EC harboring distant metastases is highly variable. Moreover, surgical resection remains the primary treatment option.

The effectiveness of stratifying the N status of patients according to the number of positive LNs was validated in previous studies. However, the validity of this method is decreased because N2 and N3 patients have similar prognoses [8–11]. Although the 7th edition of the AJCC/UICC TNM classification is based on a large database collected over a long period from 13 institutions on 3 continents, there are various factors that may display heterogeneity [12]. For example, surgical procedures, post-surgical treatments and patient follow-up practices can vary greatly between institutions. Additionally, from the 1970s to the 2000s, the treatment guidelines have been modified several times. In addition, the LN regions involved [11, 13–15] and the LN ratio (LNR) [16–18], important patterns of LN involvement in cancer, are indicators of ESCC patient survival. These findings have aroused interest in identify patterns of LN involvement that accurately predict survival. In the present study, we reviewed a large cohort of patients who had undergone potentially curative surgery with or without adjuvant therapy for ESCC to evaluate likely prognostic factors with particular emphasis on the significance of different types of LN involvement.

## Materials and Methods

### Patients and treatment

A total of 2016 ESCC cases were treated with radical resection o from 2002 to 2010. In our database, there were 688 patients with N0 (LN negative). The median OS for those patients was 31 months, and the 1-year, 3-year, and 5-year OS rates were 89.6%, 72.3%, and 62.9%, respectively. All consecutive cases of N1 primary EC between June 2002 and June 2010 treated at Zhejiang Cancer Hospital were included in this retrospective study. Examinations such as physical checkups, laboratory tests, esophagogastroduodenoscopy, barium esophagography, computerized tomography (CT), endoscopic ultrasound (EUS), spirometry and whole-body bone emission CT (ECT) were utilized to determine the preoperative clinical stage of ESCC. 18F-fluorodeoxyglucose positron emission tomography (FDG-PET) and mediastinoscopy were performed only when necessary. The following eligibility criteria were included: (1) ESCC was confirmed based on a histopathological examination; (2) the patients exhibited negative (R0) margins and survived at least 30 days postoperatively; (3) the primary tumor was located in the thoracic esophagus; (4) no malignant tumor was observed in other organs; and (5) the patients did not receive any type of induction therapy before surgery. Patients who underwent incomplete LN dissection or R1 or R2 resection were excluded. Disease stage was determined according to the TNM classification system, seventh edition [19]. The patients who were found to exhibit poor prognostic factors after surgery received further adjuvant radiotherapy or chemotherapy. Patients who experienced recurrence received further treatments. This study was approved by the Ethics and Scientific Committees of Zhejiang Province Cancer Hospital. The participants provided written informed consent for the use of their clinical records in this study.

The number of involved LNs was classified according to the 7th edition of the TNM staging system, which defined celiac axis nodes and cervical paraesophageal nodes as regional LNs regardless of the site of the primary tumor. We also categorized the LNs into four metastatic regions: cervical paraesophageal nodes, upper mediastinal nodes, middle-lower mediastinal nodes and upper abdominal nodes. Moreover, we defined the ratio of the number of involved LNs to the total number of nodes removed as the LNR. The patients were stratified into two groups according to the LNR: ≤ 0.15 and >0.15.

Generally, the patients received postoperative chest CT scans and B-scan ultrasonography of the abdomen every 3 months for 2 years, every 6 months for the next 3 years, and every year thereafter to monitor tumor recurrence. All EC patients were followed up by telephone for at least for 5 years until death or loss to follow-up. The date of the most recent follow-up was April 30, 2014. The medium follow-up duration was 56 months (range 3.1–128 months).

### Statistical analysis

OS was calculated from the date of the operation to the date of death or last living contact. Progression-free survival (PFS) was calculated from the date of operation to the date of tumor progression or recurrence, the occurrence of secondary primary tumors, or the most recent follow-up. PFS and OS were calculated using the Kaplan–Meier method, and differences between the Kaplan–Meier curves were assessed using the log-rank test for univariate survival analysis. Continuous variables were categorized based on clinical experience. Cox proportional hazard regression was used to evaluate the hazard ratios (HRs) and the 95% confidence intervals (CIs) for multivariate survival analysis. We included possible prognostic factors in the regression analysis according to clinical experience and the results of the univariate survival analysis, which were considered to be significant at a level of 0.10 or lower. Statistical significance was defined as a two-sided *P* value <0.05. All statistical analyses were performed using SPSS 18 software (SPSS Inc., Chicago, IL, USA).

## Results

### Patient characteristics

All of the 688 patients enrolled had regional LN metastases. All cases were potentially resectable (T1-4aN1-3M0) and were treated with radical resection. The surgical procedures routinely used a right thoracic approach. Most patients underwent a modern two-region lymphadenectomy, which included LN dissection of the mediastinal and bilateral recurrent laryngeal nerve chain. Of those patients, 328 (47.7%) were staged as pN1, 254 (36.9%) were staged as pN2, and 106 (15.4%) were staged as pN3.

Stage II and III disease were reported in 73 (10.6%) and 615 (89.4%) patients, respectively. Adjuvant treatment was administered to 236 (34.3%) patients. The median number of LNs resected was 28 (range 15–79), and the mean was 27.3. The mean number of metastatic LNs was 4.0 (median 3, range 1–22). A total of 236 (34.3%) patients received adjuvant radiotherapy or chemotherapy due to the observation of poor prognostic factors, recurrence or metastatic disease after operation. Among these patients, 155 received adjuvant radiotherapy (45–50.4 Gy), and 138 patients received adjuvant chemotherapy (base on cisplatin and fluoropyrimidine). In addition, 57 patients received both adjuvant radiotherapy and chemotherapy. It means that the baseline for patients with and without adjuvant radiotherapy and/or chemotherapy is not completely consistent, because the patients were involves with more advanced stage and risk prognostic factors in the latter group. The demographic characteristics, postoperative treatments and pathologic characteristics are summarized in Table 1.

**Table 1:** Baseline characteristics.

Patient characteristic	No. (%)
Sex	
Male	614 (89.2%)
Female	74 (10.8%)
Age, y	
<58	345 (50.1%)
≥58	343 (49.9%)
Smoking status	
Non-smoker	163 (23.7%)
Smoker	526 (76.5%)
Drinking status	
Non-drinker	223 (32.4%)
Drinker	465 (67.6%)
Tumor size (cm)	
≤2 cm	348 (50.6%)
>2 cm	340 (49.4%)
Pathologic T stage	
T1 or T2	105 (15.3%)
T3 or T4	583 (84.7%)
Tumor location	
Upper	13 (1.9%)
Middle	282 (41.0%)
Lower	393 (57.1%)
Tumor differentiation	
Well	75 (10.9%)
Intermediate	475 (69.1%)
Poor or undifferentiated	137 (19.9%)
Lymph node metastasis (2009)	
N1	328 (47.7%)
N2	254 (36.9%)
N3	106 (15.4%)
Involved LNR	
>0.15	244 (35.5%)
≤0.15	444 (64.5%)
Clinical stage	
IIB	73 (10.6%)
IIIA	270 (39.2%)
IIIB	233 (33.9%)
IIIC	112 (16.3%)
Venous/lymphatic invasion	
No	482 (70.1%)
Yes	206 (29.9%)
Perineural invasion	
No	468 (68.0%)
Yes	220 (32.0%)
No. of LN regions involved	
One region	339 (51.8%)
Two regions	246 (37.6%)
Three or four regions	69 (10.6%)
Adjuvant radiotherapy or chemotherapy	
No	452 (65.7%)
Yes	236 (34.3%)

Abbreviations: LN = lymph node; LNR = lymph node ratio

Direct and metastatic LN involvement was observed in 32 patients in the cervical paraesophageal LN region, in 223 patients in the upper mediastinal LN region, in 384 patients in the middle-lower mediastinal LN region and in 401 patients in the upper abdominal LN region. More patients exhibited metastases in the upper abdominal LNs, likely because more tumors (57.1%) were located in the lower esophagus. Of all patients, 315 harbored metastatic LNs in more than 2 regions.

### Univariate and multivariate analysis of survival

**Fig 1:**
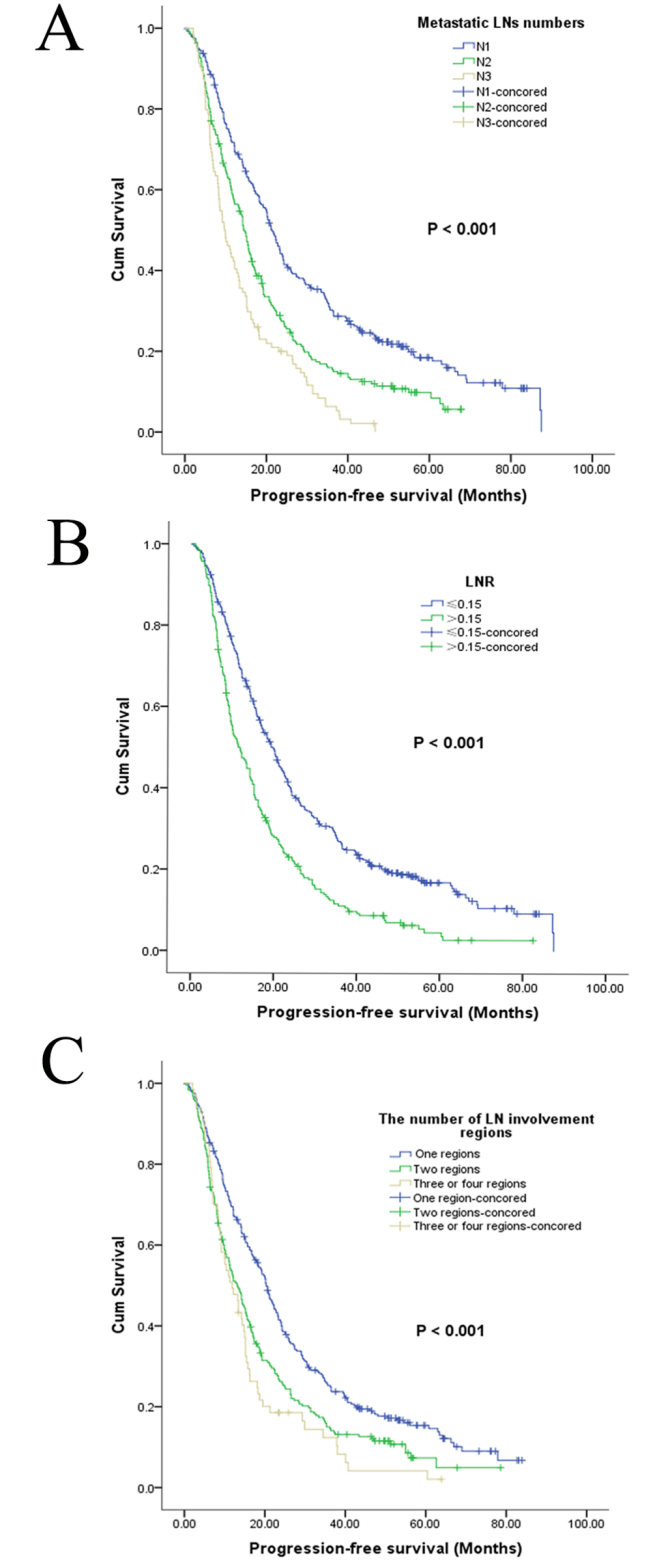
Kaplan–Meier progression-free survival curves for 668 patients. The patients were stratified by the number of metastatic lymph nodes (A), the lymph node ratio (B), or the number of involved lymph node regions (C).

**Fig 2:**
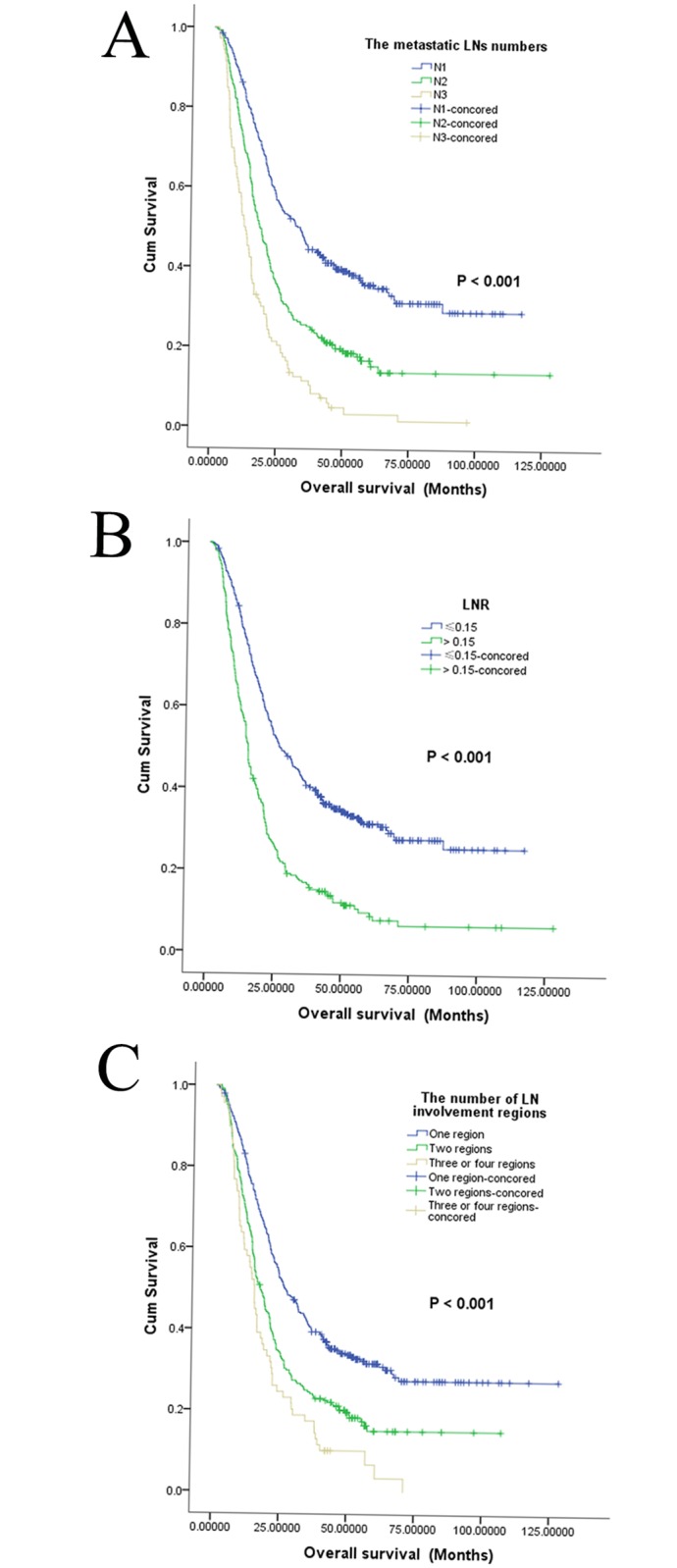
Kaplan-Meier overall survival curves for 668 patients. The patients were stratified by the number of metastatic lymph nodes (A), the lymph node ratio (B), or the number of involved lymph node regions (C).

In the entire LN positive cohort, the median PFS was 16.6 months, and the 1-year, 3-year, and 5-year PFS rates were 64.4%, 19.2%, and 8.6%, respectively. Additionally, the median OS was 21.3 months, and the 1-year, 3-year, and 5-year OS rates were 74.0%, 72.3%, and 23.4%, respectively. Cancer confined to a single region was associated with better outcomes than cancer distributed among multiple regions (*P* < 0.001 for both PFS and OS) (Figs 1 and 2, Table 2). In addition, an increased number of metastatic LNs was significantly associated with poorer PFS and OS (Figs 1 and 2) based on univariate analysis (*P* < 0.001). PFS and OS were significantly higher in patients with a lower LNR (Figs 1 and 2), with 5-year PFS rates of 16.8% and 4.5% (*P* < 0.001) and 5-year OS rates of 31.4% and 9.7% for patients with lower and higher LNR, respectively (*P* < 0.001). Factors such as sex, smoking status, tumor differentiation, tumor location, T status, clinical stage, venous/lymphatic invasion and perineural invasion were found to influence PFS based on univariate analysis. Sex, smoking status, drinking status, tumor size, tumor differentiation, tumor location, T status, clinical stage, venous/lymphatic invasion and perineural invasion were statistically significantly associated with OS based on univariate analyses (Table 2). No difference in either PFS or OS was detected between patients with and without adjuvant radiotherapy or chemotherapy.

**Table 2:** Univariate analysis of the clinical factors.

Patient Characteristics	HR (95% CI) for PFS	P Value	HR (95% CI) for OS	P Value
Sex				
Male	22.1%	**0.015**	37.5%	**0.007**
Female	10.3%		22.1%	
Age, y				
<58y	13.2%	0.487	26.2%	0.377
≥58y	9.0%		21.6%	
Smoking status				
Non-smoker	15.5%	**0.017**	31.7%	**0.003**
Smoker	10.5%		21.2%	
Drinking status				
Non-drinker	12.7%	0.089	28.4%	**0.041**
drinker	10.3%		21.4%	
Tumor size (cm)				
<5cm	10.1%	0.122	19.4%	**0.000**
≥5cm	12.6%		28.1%	
Pathologic T stage				
T1+T2	22.6%	**0.000**	38.7%	**0.000**
T3+T4	9.4%		21.0%	
Tumor location				
Middle+Upper	13.0%	**0.041**	21.7%	**0.007**
Lower	10.1%		26.5%	
Differentiation				
well	25.2%	**0.002**	40.5%	**0.000**
Intermediate	11.1%		23.8%	
Poor or undifferentiated	7.7%		12.9%	
Lymph node metastasis (2009)				
N1	18.4%	**0.000**	35.7%	**0.000**
N2	9.8%		16.7%	
N3	0.0%		3.3%	
Involved LNR				
>0.15	4.5%	**0.000**	9.7%	**0.000**
≤0.15	16.8%		31.4%	
Venous/lymphatic invasion				
No	13.8%	**0.006**	26.0%	**0.005**
Yes	8.5%		17.3%	
Perineural invasion				
No	13.6%	**0.015**	26.9%	**0.000**
Yes	9.2%		16.9%	
No. of LN involved				
One area	15.4%	**0.000**	31.7%	**0.000**
Two areas	7.4%		15.1%	
Three or four areas	4.1%		13.0%	
Adjuvant radiotherapy or chemotherapy				
No	11.0%	0.708	23.7%	0.596
Yes	13.0%		23.7%	

Abbreviations: PFS = Progression-free survival; OS = Overall survival.

Variables displaying a P value of < 0.05 based on univariate analyses were included in the multivariate analysis. Patients with N1 LN involvement experienced longer survival than patients with N2 (HR: 1.37; 95% CI: 1.12–1.68; *P* < 0.001) or N3 LN involvement (HR: 1.96; 95% CI: 1.52–2.53). Additionally, patients with N1 LN involvement exhibited a higher pathologic T stage. Those with an increased LNR exhibited longer OS than those with a lower LNR based on multivariate analysis (HR: 1.45; 95% CI: 1.15–1.84; *P* = 0.002). Similarly, tumor location, clinical stage and perineural invasion were significant prognostic factors for OS (Table 3). Moreover, age, gender and tumor differentiation served as independent prognostic factors for both PFS and OS.

**Table 3:** Multivariate analyses of various factors affecting progression-free survival and overall survival using a cox proportional hazards model.

Factor	Progression-free survival			Overall survival		
	Hazard ratio	95% CI	*P* value	Hazard ratio	95% CI	*P* value
Age, y						
<58	Ref.			Ref.		
≥58	1.19	0.99–1.43	0.052	1.25	1.04–1.50	0.015
Smoking status						
Non-smoker	Ref.			Ref.		
Smoker	1.32	1.06–1.65	0.013	1.25	1.04–1.50	0.050
Pathologic T stage						
T1 or T2	Ref.					
T3 or T4	1.42	1.08–1.85	0.011			
Tumor location						
Lower				Ref.		
Middle or upper				0.83	0.69–0.99	0.043
Differentiation						
Well	Ref.		0.012	Ref.		0.002
Intermediate	1.26	0.91–1.75	0.157	1.33	0.96–1.84	0.085
Poor or undifferentiated	1.65	1.14–2.37	0.007	1.81	1.26–2.60	0.001
Lymph node metastasis						
N1	Ref.		< 0.001			
N2	1.37	1.12–1.68	0.002			
N3	1.96	1.52–2.53	< 0.001			
Involved LNR						
≤0.15				Ref.		
>0.15				1.45	1.15–1.84	0.002
Clinical stage						
IIB				Ref.		< 0.001
IIIA				1.19	1.19–1.69	0.347
IIIB				1.67	1.17–2.39	0.005
IIIC				2.15	1.40–3.29	< 0.001
Perineural invasion						
No				Ref.		
Yes				1.29	1.07–1.56	0.008

Furthermore, we conducted a subgroup analysis to assess whether different regions of LN involvement, the LNR, and the number of metastatic LNs could be utilized as independent prognostic factors for PFS and OS in patients with or without postoperative treatments. The results did not differ from those for the entire cohort (data not shown).

## Discussion

LN-related factors including the number of LN regions involved, the LNR, and the number of metastatic LNs are strong prognostic indicators for ESCC patients. Thus, these factors often influence decisions about the timing and selection of treatments. N descriptors, which are defined according to the number of metastatic LNs, were significantly altered in the new version of the TNM system. Growing evidence indicates that the number of positive LNs positively correlates with prognosis [20–23]. However, most of these studies were based on retrospective analysis of pathologic data from patients treated with surgery alone. The role of various regions of LN involvement and the LNR has not been well characterized to date. Identifying optimal patient subgroups according to LN-related factors might help physicians select the most appropriate postoperative management strategies. In this study, we evaluated prognostic factors, particularly examining the prognostic significance of different types of LN involvement in ESCC patients. To the best of our knowledge, this is the largest retrospective study performed to date.

Previous studies [20–25] have suggested that survival is significantly worse in patients exhibiting increased LN involvement; however, other studies have found no significant differences between the N2 and N3 subgroups [6, 8–11, 15]. Thus, the clinical implications of the number of LNs involved in N-positive ESCC remain controversial. On the one hand, Wang et al [3] analyzed data from the Worldwide Esophageal Cancer Collaboration database, which included 4,627 patients with ESCC. They concluded that the number of metastatic LNs is an independent prognostic factor for survival in patients with ESCC. On the other hand, Yamasaki et al [6] reported that no significant differences in survival were detected between the N2 and N3 subgroups, with an HR of 1.31 (CI: 0.740–2.18; *P* = 0.340). In the present study, the number of metastatic LNs was an independent predictor of survival in ESCC, and this finding is consistent with those of previous studies [20–24].

The number of regions of LN involvement has also been recognized as a significant predictor of survival in ESCC. Previous studies have shown that the involvement of multiple LN nodules or stations may indicate poorer prognosis than the involvement of a single LN nodule [11, 13–15]. We also found a strong correlation between the number of regions of LN involvement and survival. The number of involved LN regions might provide a more reliable approach because the number of metastatic LNs may be overestimated due to damage caused by surgical procedures or underestimated based on an incomplete pathologic examination.

The association between survival and the number of metastatic LNs is influenced by the number of LNs removed. The LNR, which is considered to illustrate the metastatic LN status more accurately than the number of metastatic LNs, is found to have an impact on the prognosis of ESCC patients. In patients undergoing esophagectomy without preoperative chemoradiation, the National Comprehensive Cancer Network (NCCN) guidelines recommend that at least 15 LNs be removed for adequate nodal staging [16]. We restricted our analysis to the recommended extent of LN dissection [16]. Currently, there is no consensus regarding the optimal cut-off value for the LNR. In a study reported by He et al [17], the LNR was a prognostic factor for OS based on univariate analysis. Patients with a LNR < 0.15, of 0.15–0.30, or > 0.30 experienced 5-year OS rates of 30.1%, 17.8%, and 9.5%, respectively (*P* < 0.001). Wang et al [18] reported a dataset of 209 resected patients with ESCC. Node-positive patients displaying a LNR greater than 0.2 exhibited significantly poorer PFS (*P* = 0.008, HR 1.86, 95% CI 1.18–2.94) and OS (*P* = 0.025, HR 1.71, 95% CI 1.07–2.73). However, this study included patients with stage I ESCC. Our study also showed that a LNR greater than 0.15 was an independent prognostic factor for both PFS and OS in EC patients. Moreover, the LNR displayed greater prognostic value than the N stage for OS of ESCC patients based on multivariate analysis. Prospective multicenter studies are needed to validate this result.

We acknowledge that our study has several limitations. First, our study utilized a retrospective design and was conducted at a single institution. Second, the study group was heterogeneous and included patients who received adjuvant therapy after surgical resection, patients who were treated under different treatment guidelines between 2002 and 2010, and patients who underwent LN dissection using different methods (two or three fields). The large number of patients included in this study may weaken these potential biases. Although the current NCCN guidelines [16] recommended that induction chemoradiation followed by surgical resection is the optimal treatment for pN+ ESCC patients, adjuvant treatment was conducted in only 34.3% patients in this study. Besides, there was no difference in either PFS or OS between patients with and without adjuvant radiotherapy and/or chemotherapy (Table 2), possibly because patients who were <65 years old, had a tumor size >5 cm or exhibited other risk factors were more likely to receive postoperative adjuvant therapy. It means that the baseline for patients with and without adjuvant radiotherapy and/or chemotherapy is not completely consistent, because the patients were involves with more advanced stage and risk prognostic factors in the latter group. Third, preoperative staging was not sufficiently accurate, and few patients had undergone PET-CT.

In conclusion, our study has shown that not only the number of metastatic LNs but also the LNR can predict outcome after definitive surgery among Chinese patients with N-positive ESCC. Moreover, the number of involved LN regions serves as a potential prognostic factor. The LNR was a stronger prognostic factor of OS than the number of LN metastases based on multivariate analysis. Thus, the LNR might serve as a powerful factor that should be included in TNM staging for EC patients. In addition, the number of involved LN regions and the LNR may be used to stratify patients into subgroups with a distinct risk for recurrence. pT status, tumor location, clinical stage and perineural invasion were also demonstrated as independent prognostic factors. Nonetheless, additional studies will be required to confirm our findings, and the cutoff value for the LNR must be further defined.
